# The Combination of Electroacupuncture and Massage Therapy Alleviates Myofibroblast Transdifferentiation and Extracellular Matrix Production in Blunt Trauma-Induced Skeletal Muscle Fibrosis

**DOI:** 10.1155/2021/5543468

**Published:** 2021-07-07

**Authors:** Na Zhao, Bo Liu, Si-Wen Liu, Wei Zhang, Hua-Nan Li, Geng Pang, Xiong-Fei Luo, Jin-Gui Wang

**Affiliations:** ^1^First Teaching Hospital of Tianjin University of Traditional Chinese Medicine, National Clinical Research Center for Chinese Medicine Acupuncture and Moxibustion, Tianjin 300193, China; ^2^Tianjin University of Traditional Chinese Medicine, Tianjin 301617, China

## Abstract

Complementary therapies, such as acupuncture and massage, had been previously reported to have therapeutic effects on skeletal muscle contusions. However, the recovery mechanisms on skeletal muscles after blunt trauma via the combination of electroacupuncture (EA) and massage therapy remain unclear. In the present study, a rat model of the skeletal muscle fibrosis following blunt trauma to rat skeletal muscle was established, and the potential molecular mechanisms of EA + massage therapy on the skeletal muscle fibrosis were investigated. The results suggested that EA + massage therapy could significantly decrease inflammatory cells infiltration and collagenous fiber content and ameliorate the disarrangement of sarcomeres within myofibrils compared to the model group. Further analysis revealed that EA + massage therapy could reduce the degree of fibrosis and increase the degree of myofibroblast apoptosis by downregulating the mRNA and protein expression of transforming growth factor- (TGF-) *β*1 and connective tissue growth factor (CTGF). Furthermore, the fibrosis of injured skeletal muscle was inhibited after treatment through the normalization of balance between matrix metalloproteinase- (MMP-) 1 and tissue inhibitor of matrix metalloproteinase (TIMP). These findings suggested that the combination of electroacupuncture and massage therapy could alleviate the fibrotic process by regulating TGF *β*1-CTGF-induced myofibroblast transdifferentiation and MMP-1/TIMP-1 balance for extracellular matrix production.

## 1. Introduction

Skeletal muscle injury is one of the common types of exercise-related injuries in sports medicine, and its effective treatment is rather challenging. The regeneration process of skeletal muscle injury is similar in most types of muscle injuries. However, it is usually hard to achieve complete recovery from the injury because of the development of fibrosis in the second week after the injury. The formed scar tissue is usually difficult to exert the normal muscle fiber functions and more susceptible to be reinjured [1]. Surgery is a treatment option only implemented in certain specific conditions, such as the presence of a large intramuscular hematoma, a complete strain or tear of a muscle with few agonist muscles, or a partial strain with persistent extension pain (>4–6 months) [2]. Conservative treatment strategies are frequently used for acute and chronic skeletal muscle injuries [3], which aim to minimize further damage, relieve pain, reduce hemorrhage and edema, and promote healing. For example, physical therapy approaches such as limb elevation and local cooling had been used to improve muscle repair [4]. Another alternative treatment of enhancing muscle healing includes pharmacological therapies with nonsteroidal anti-inflammatory drugs (NSAIDs) being the most common drug [5]. Although the NSAIDs could reduce the early inflammatory responses, there were some side effects such as hypertension, gastrointestinal disorders, and renal toxicity. Recently, complementary therapies such as acupuncture and massage therapy had gained increasing attention for the treatment of skeletal muscle injuries [6].

Acupuncture, one branch of traditional medicine, is widely used to treat various diseases in clinics [7]. The World Health Organization (WHO) had issued a consensus statement that current data supported the use of acupuncture to treat certain diseases, such as the stroke rehabilitation, low back pain, headache, carpal tunnel syndrome, osteoarthritis, asthma, and other conditions [8]. Furthermore, electroacupuncture (EA) is usually used to recover musculoskeletal disorders and skeletal muscle fatigue [9, 10]. Massage therapy mainly involves topical stroking and kneading of the skin and underlying musculature to produce pressure and muscle distension. Massage is widely used to treat moderate muscle injuries for reducing muscle soreness and enhancing postexercise muscle recovery [11]. Although massage has become a common complementary treatment approach in muscle repair, its potential mechanisms are still unclear.

Skeletal muscle injury contains three main phases including inflammation, regeneration, and fibrosis [12]. Skeletal muscle fibrosis is characterized by the redundant accumulation of matrix, unbalance of synthesis, and the degradation of matrix proteins. Transforming growth factor-*β* (TGF-*β*) and connective tissue growth factor (CTGF) are two potent profibrotic growth factors that induce the matrix production and accumulation. Moreover, CTGF is the downstream effector of TGF-*β*1 [13]. Matrix metalloproteinases (MMPs) played vital roles in maintaining the functional integrity of myofiber. The extracellular matrix (ECM) can be broken down by MMPs to allow cell growth, and skeletal muscle cell migration and differentiation are also regulated by MMPs [14]. The MMP collagenases, such as MMP-1, -8, and -13, show the ability to clear the interstitial collagen types I, II, and III, and MMP-2 and -9 can degrade the denatured collagen [15].

In this study, a rat model of the skeletal muscle fibrosis following blunt trauma to rat skeletal muscle was established, and the effects of the combination of EA and MST and the possible mechanisms on the skeletal muscle fibrosis were investigated.

## 2. Materials and Methods

### 2.1. Reagents

PrimeScript™ RT Master Mix kit was bought from TaKaRa Co. (Kyoto, Japan). The cell lysis kit, RNA extraction kit, and anti-GAPDH were purchased from Sangon Biotech (China). Anti-TGF-*β*1, anti-CTGF, and anti-TIMP-1 were obtained from Abcam (United Kingdom). Anti-Smad 2 and anti-Smad 3 were obtained from CST (USA). Anti-MMP-1 was obtained from Thermo Fisher Scientific (USA).

Sprague-Dawley (SD) rats (male, 180–220 g) were obtained from Beijing Vital River Laboratories Animal Technology Co., Ltd. Experiments were performed in accordance with the NIH Guide for the Care and Use of Laboratory Animals and approved by the Institute Ethics Committee (TCM-2019-013-E04, Tianjin University of Traditional Chinese Medicine).

### 2.2. Model of Skeletal Muscle Fibrosis and Treatment in Rats

A rat model of the skeletal muscle fibrosis following blunt trauma to rat skeletal muscle was established by the method of Kami et al. [16] with minor modifications. The rats were allocated into 5 groups randomly (*n* = 16/group): control group, skeletal muscle fibrosis model group, skeletal muscle fibrosis model group + treatment with massage therapy group, skeletal muscle fibrosis model group + treatment with electroacupuncture therapy group, and skeletal muscle fibrosis model group + treatment with the combination of massage and electroacupuncture therapy. The massage therapy was performed based on our previous study [17]. In brief, the massage was performed with the massage manipulation simulator on the damaged tissue for 10 minutes per day and continued with a total intervention time of 25 days. EA was performed based on the previous method [18]. Rats were performed with EA at Zusanli with synchronously stimulated identical parameters for 15 min, which was continued for a total of 25 days.

### 2.3. Histological Assessment and Masson's Trichrome Stain

After the treatment of massage therapy and electroacupuncture therapy, rats were sacrificed by anaesthetization with pentobarbital sodium. The skeletal muscle was then separated quickly on ice. The samples were fixed in 10% buffered formalin solution and dehydrated by ethanol and xylene. After fixation and dehydration, the samples were then embedded in paraffin and cut into sections with 5 *μ*m thickness. The obtained sections were stained with H&E and Masson's trichrome stain and observed under the light microscope.

### 2.4. Immunofluorescence of *α*-SMA and TUNEL Staining

The expression of *α*-SMA was analyzed by the immunofluorescence staining using the *α*-SMA antibody and the immunofluorescence detection kit. In brief, slides of skeletal muscle were deparaffinized and rehydrated by using xylene, ethanol, and water, sequentially. After pairing the retrieval antigen and blocking the nonspecific antigen in skeletal muscle tissue, the tissues were incubated overnight with a dilution of anti-*α*-SMA antibody at 4°C. The tissues were then incubated with diluted fluorescent-conjugated secondary antibody for 1 h at room temperature. After incubation, the secondary antibody was washed off, and TUNEL staining was performed based on the manufacturer's instructions. Finally, tissues were incubated with DAPI staining solution for 5 minutes. Fluorescence was observed using a laser confocal microscope.

### 2.5. Preparation of Muscle Samples for Transmission Electron Microscopy (TEM) Analysis

For the TEM analysis, the muscle samples were cut into 3 mm cubes and fixed for 4 h in cold 2.5% glutaraldehyde at 4°C. The muscle tissues were then dehydrated with ethanol and acetone. The dehydrated tissues were embedded in epoxy resin and cut into sections with 2 *μ*m thickness. The toluidine blue was used to stain the specific area, which was further cut into 50 nm thickness collected to a copper grid. The ultrathin section was stained with uranyl acetate to acquire the ultrastructure using the Hitachi TEM system (Tokyo, Japan).

### 2.6. Real-Time PCR

RNA from muscle tissues was extracted using a Trizol extraction kit. The reverse transcription was performed with PrimeScript RT Master Mix at 37°C for 15 min, 85°C for 5 sec, 4°C. Finally, PCR amplification was performed using TB Green™ Premix Ex Taq™ II. The oligonucleotide primers are shown in Table S1. The amplification was carried out by a 7500 real-time PCR system (ABI Prism) with protocols of TB Green™ Premix Ex Taq™ kits. At the end of each experiment, a melt curve analysis was performed. The mRNA expression was analyzed using the 2^−ΔΔCT^ method [19, 20].

### 2.7. Western Blot

The protein expression levels of TGF-*β*, CTGF, MMP-1, and TIMP-1 were investigated using the western blot method. The total protein was extracted and separated on a 10% SDS-polyacrylamide gel and electroblotted to a PVDF membrane. The PVDF membrane was firstly incubated with 5% skim milk for 2 h, and then the PVDF membrane was incubated with anti-TGF-*β*1, anti-CTGF, anti-MMP-1, anti-TIMP-1, and anti-GAPDH antibody overnight at 4°C. The obtained blots were washed with TBS and incubated with horseradish peroxidase-conjugated secondary antibody. Then, the blots were washed with TBS again and incubated with horseradish peroxidase substrate for 5 minutes. At last, the protein expression was obtained on a ChemiScope 6200 Chemiluminescence imaging system.

### 2.8. Statistical Analysis

The data were analyzed with Origin Pro 9.0 software. The results are presented as the mean ± SD. Statistically significant differences between values of different groups were determined with one-way ANOVA following by Scheffe's multiple range test. The value of *P* < 0.05 was considered statistically significant.

## 3. Results

### 3.1. Effect of the Combination of Massage and Electroacupuncture Therapy on Histological Change and the Degree of Fibrosis in the Skeletal Muscle Fibrosis Rats

**Figure 1:**
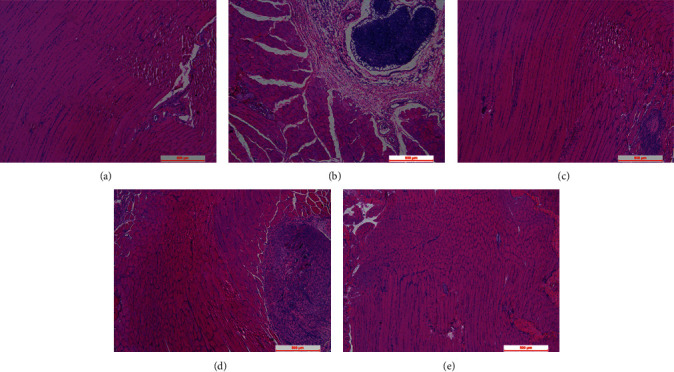
Histological recovery of skeletal muscle of a rat model with the skeletal muscle fibrosis. Histological sections with hematoxylin and eosin (HE) staining of skeletal muscle in rat. (a) Rat treated without blunt trauma to skeletal muscle (control group). (b) Rat treated after blunt trauma to skeletal muscle (model group). (c) Rat treated with massage therapy after blunt trauma to skeletal muscle (massage therapy group). (d) Rat treated with electroacupuncture therapy after blunt trauma to skeletal muscle (electroacupuncture therapy group). (e) Rat treated with the combination of continuous massage and electroacupuncture therapy after blunt trauma to skeletal muscle (the combination treatment group). Scale bars, 500 *μ*m.

**Figure 2:**
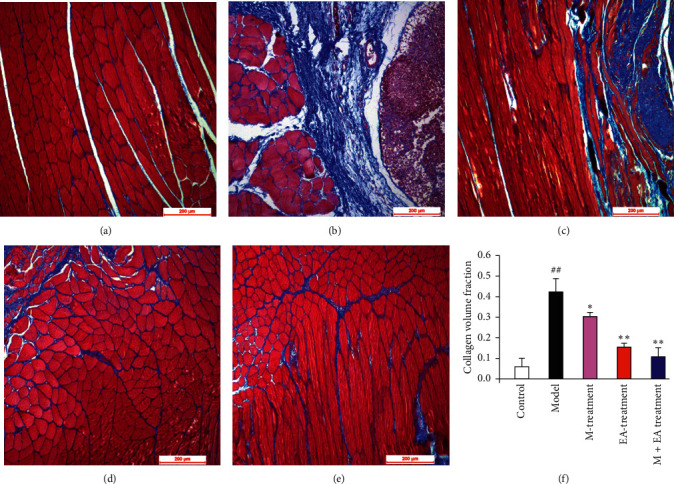
Fibrosis in skeletal muscle of blunt trauma-induced rats. Histological sections with Masson's trichrome staining of skeletal muscle in rats. (a) Rat treated without blunt trauma to skeletal muscle (control group). (b) Rat treated after blunt trauma to skeletal muscle (model group). (c) Rat treated with massage therapy after blunt trauma to skeletal muscle (massage therapy group). (d) Rat treated with electroacupuncture therapy after blunt trauma to skeletal muscle (electroacupuncture therapy group). (e) Rat treated with the combination of continuous massage and electroacupuncture therapy after blunt trauma to skeletal muscle (the combination treatment group). Scale bars, 200 *μ*m. (f) Collagen volume fraction of the skeletal muscle. ^#^*P* < 0.05 and ^##^*P* < 0.01 vs. control. ^*∗*^*P* < 0.05 and ^*∗∗*^*P* < 0.01 (*n* = 4) vs. model.

The results of H&E staining are shown in Figure 1. The myofibrous tissue of normal rats showed polygonal, regular, and tight distribution, and no edema, hyperaemia, and inflammation were observed (Figure 1(a)). In the model of the skeletal muscle fibrosis group, the skeletal muscle cells tend to be round, and there were more inflammatory cells infiltrating in damaged skeletal muscle than those in the normal cells (Figure 1(b)). The condition was improved by continuous massage therapy or electroacupuncture therapy with fewer inflammatory cells surrounding the damaged skeletal muscle tissue (Figures 1(c) and 1(d)). No inflammatory cell infiltration was observed in the treatment group with the combination of continuous massage and electroacupuncture therapy, and the shape of skeletal muscle cells was similar to the control group (Figure 1(e)). As shown in Figure 2(f), collagenous fiber volume fraction of the massage therapy group, electroacupuncture therapy group, and the combination treatment group significantly reduced than the model group (*P* < 0.01). Among three groups, the combination treatment group is the least (*P* < 0.05 or *P* < 0.01). Collagenous fibers are a major component of the ECM. Also, they are the most abundant protein within the body. Therefore, the result indicated the combination treatment group is effective to reduce ECM production in blunt trauma-induced skeletal muscle fibrosis.

Masson's trichrome stain was used for evaluating the degree of fibrosis for the skeletal muscle (Figure 2). There was little collagenous fiber in the control group (Figure 2(a)), but the content of collagenous fiber increased significantly in the model of the skeletal muscle fibrosis group (Figure 2(b)). The content of collagenous fiber decreased after continuous massage therapy or electroacupuncture therapy (Figures 2(c) and 2(d)), and the combination of continuous massage and electroacupuncture therapy showed least content of collagenous fiber (Figures 2(e) and 2(f)).

### 3.2. Effect of the Combination of Massage and Electroacupuncture Therapy on Apoptosis of Myofibroblast in the Skeletal Muscle Fibrosis Rats

**Figure 3:**
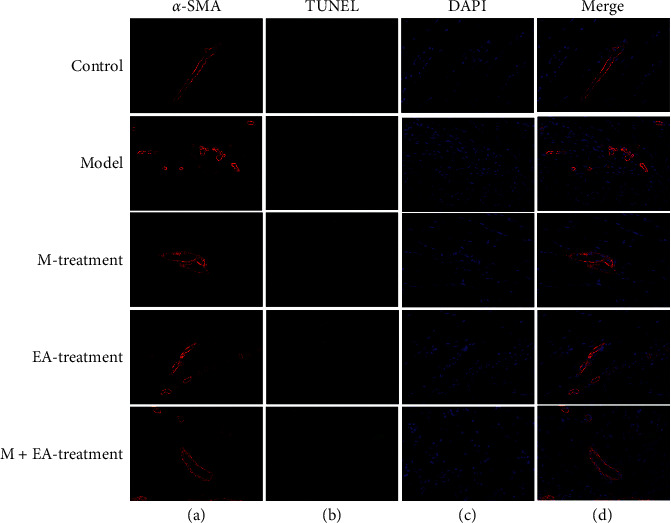
The apoptosis of myofibroblast in the skeletal muscle fibrosis rats. (a) Rat treated without blunt trauma to skeletal muscle (control group). (b) Rat treated after blunt trauma to skeletal muscle (model group). (c) Rat treated with massage therapy after blunt trauma to skeletal muscle (massage therapy group). (d) Rat treated with electroacupuncture therapy after blunt trauma to skeletal muscle (electroacupuncture therapy group). (e) Rat treated with the combination of continuous massage and electroacupuncture therapy after blunt trauma to skeletal muscle (the combination treatment group). The orange was *α*-SMA, and the DAPI (blue) was used to stain the cell nucleus. The apoptosis of myofibroblast was observed with the TUNEL assay kit (green).

The apoptosis of myofibroblast was investigated by immunofluorescent staining (Figure 3). The myofibroblast was firstly marked with *α*-SMA (red), and the TUNEL was used to examine the apoptosis of myofibroblast (green). Compared with the control group, the fluorescence intensity of *α*-SMA (red) was increased, but the apoptosis of myofibroblast (green) was not increased. After the continuous massage therapy or electroacupuncture therapy, the positive fluorescence intensity (red) of *α*-SMA was decreased. However, the apoptosis of myofibroblast (green) was increased relatively compared to the model group. The combination of continuous massage and electroacupuncture therapy showed the most degree of apoptosis of myofibroblasts (green).

### 3.3. Effect of the Combination of Massage and Electroacupuncture Therapy on the Ultrastructure Alteration of Skeletal Muscle in the Skeletal Muscle Fibrosis Rats

**Figure 4:**
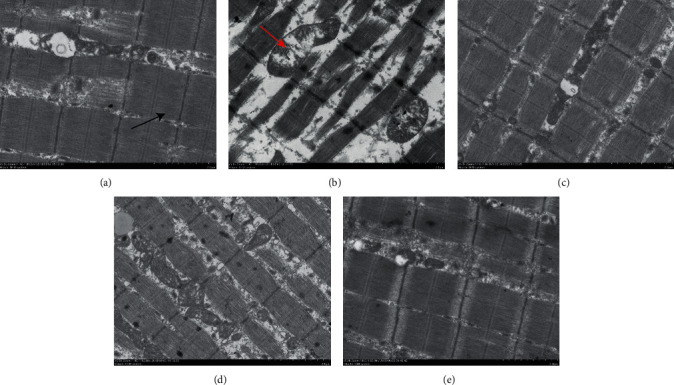
The ultrastructure alteration of skeletal muscle in the skeletal muscle fibrosis rats. (a) Rat treated without blunt trauma to skeletal muscle (control group). (b) Rat treated after blunt trauma to skeletal muscle (model group). (c) Rat treated with massage therapy after blunt trauma to skeletal muscle (massage therapy group). (d) Rat treated with electroacupuncture therapy after blunt trauma to skeletal muscle (electroacupuncture therapy group). (e) Rat treated with the combination of continuous massage and electroacupuncture therapy after blunt trauma to skeletal muscle (the combination treatment group). The sarcomeres were marked with black arrows, and the mitochondria were marked with red arrows.

The ultrastructure alteration of skeletal muscle was observed by transmission electron microscopy. In the control group, the shape of mitochondria was round and oval, and the arrangement of sarcomeres in one myofibril was relatively regular (black arrow) (Figure 4(a)). In the rat model of the skeletal muscle fibrosis, some myofibrils in the muscle were ruptured and interlaced, and the mitochondria were swollen (red arrow). Furthermore, the direction of myofilaments in one myofibril was changed (Figure 4(b)). The continuous massage therapy or electroacupuncture therapy could improve the condition compared to the model group (Figures 4(c) and 4(d)). Furthermore, after the combination of continuous massage and electroacupuncture therapy, mitochondria were shown with round and oval shape, and the arrangement of sarcomeres within one myofibril was relative orderliness, which was close to the control group (Figure 4(e)).

### 3.4. Effect of the Combination of Massage and Electroacupuncture Therapy on mRNA Expression of TGF-*β*1, CTGF, MMP-1, and TIMP-1 in the Skeletal Muscle Fibrosis Rats

**Figure 5:**
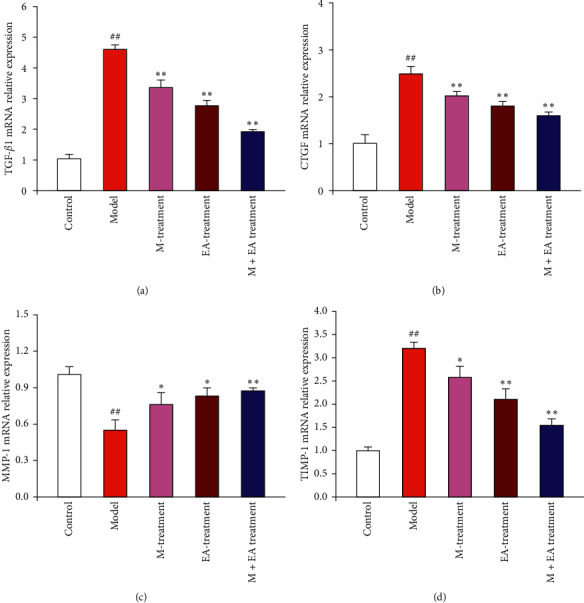
Effect of different treatments on mRNA expression of TGF-*β*1, CTGF, MMP-1, and TIMP-1 in the skeletal muscle fibrosis rats. The mRNA expression of TGF-*β*1 (a), CTGF (b), MMP-1 (c), and TIMP-1 (d) was analyzed by real-time PCR normalized to GAPDH. ^#^*P* < 0.05 and ^##^*P* < 0.01 vs. control. ^*∗*^*P* < 0.05 and ^*∗∗*^*P* < 0.01 (*n* = 4) vs. model.

For the purpose of investigating the possible mechanism of combination of massage and electroacupuncture therapy on regulating skeletal muscle fibrosis, the mRNA expressions of TGF-*β*1, CTGF, MMP-1, and TIMP-1 were firstly detected by real-time PCR (Figure 5). Compared with the control group, the mRNA expressions of TGF-*β*1 and CTGF were upregulated significantly in the model group, and the continuous massage therapy or electroacupuncture therapy could significantly downregulate the mRNA expression of TGF-*β*1 and CTGF (*P* < 0.01) (Figures 5(a) and 5(d)). The combination of continuous massage and electroacupuncture therapy had the strongest downregulation effect on the mRNA expression of TGF-*β*1 and CTGF. The mRNA expression of MMP-1 was significantly downregulated in the model group compared with the control group (*P* < 0.01), and the continuous massage therapy or electroacupuncture therapy and the combination of continuous massage and electroacupuncture therapy could upregulate the mRNA expression of MMP-1 significantly (*P* < 0.05 or *P* < 0.01) (Figure 5(c)). Moreover, the continuous massage therapy or electroacupuncture therapy could significantly downregulate the mRNA expression of TIMP-1 compared with the model group (*P* < 0.05 or *P* < 0.01), and the combination of continuous massage and electroacupuncture therapy exhibited the strongest downregulation effect on the mRNA expression of TIMP-1 (Figure 5(d)).

### 3.5. Effect of the Combination of Massage and Electroacupuncture Therapy on Protein Expression of TGF-*β*1, CTGF, MMP-1, and TIMP-1 in the Skeletal Muscle Fibrosis Rats

**Figure 6:**
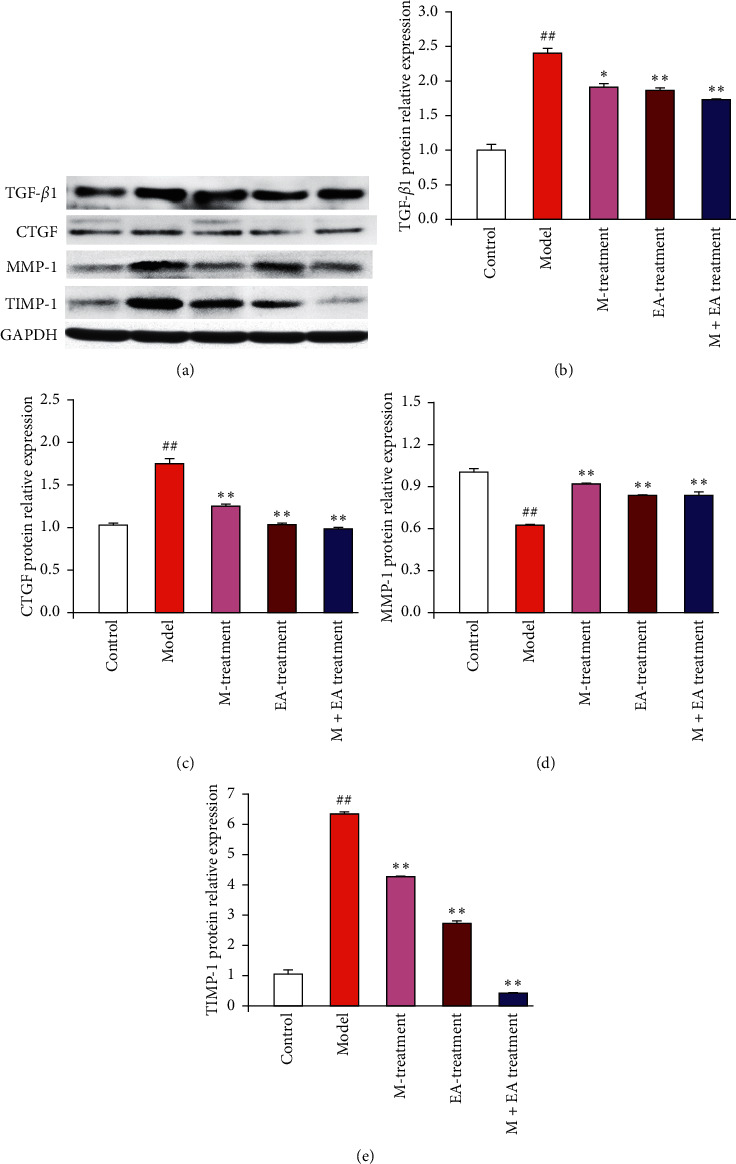
Effect of different treatments on protein expression of TGF-*β*1, CTGF, MMP-1, and TIMP-1 in the skeletal muscle fibrosis rats. The protein expression of TGF-*β*1, CTGF, MMP-1, and TIMP-1 (a) was investigated by western blot. The intensity (b–e) was quantified by ImageJ version 1.51n. ^##^*P* < 0.01 vs. control. ^*∗*^*P* < 0.05 and ^*∗∗*^*P* < 0.01 (*n* = 4) vs. model.

The balance between ECM production and degradation is mediated by MMPs and TIMPs [21]. MMP collagenases, such as MMP-1, -8, and -13, show the ability to clear the interstitial collagen types I, II, and III. TIMP-1 is an important regulator in the synthesis and degradation of ECM. The protein expressions of TGF-*β*1, CTGF, MMP-1, and TIMP-1 were investigated by western blot (Figure 6(a)). The density analysis of TGF-*β*1, CTGF, MMP-1, and TIMP-1 was obtained in Figures 6(b)–6(e). Compared with the model group, continuous massage therapy or electroacupuncture therapy and the combination of continuous massage and electroacupuncture therapy could downregulate the protein expressions of TGF-*β*1, CTGF, and TIMP-1 and significantly upregulated the protein expression of MMP-1 (*P* < 0.01). The combination of continuous massage and electroacupuncture therapy showed the strongest downregulation effect on protein expressions of TGF-*β*1, CTGF, and TIMP-1. Therefore, those results indicated the combination treatment group is effective to reduce ECM production in blunt trauma-induced skeletal muscle fibrosis by downregulating the expression of TGF-*β*1, CTGF and TIMP-1 and upregulating the expression of MMP-1.

## 4. Discussion

Previous evidence had showed that electroacupuncture treatment is safe and effective for certain diseases such as knee pain, whiplash injury, tendinitis, and dysmenorrhea [22–25]. As another alternative therapy, massage was also a widely used treatment solution for moderate muscle injuries and muscle recovery [26]. Although he effect of electroacupuncture therapy or massage therapy on contusion injury in skeletal muscle had been reported previously, the possible mechanisms of the combination of massage and electroacupuncture therapy in the recovery of skeletal muscles were still unclear.

In the present study, the combination of massage and electroacupuncture therapy was performed to treat blunt trauma in skeletal muscles. We firstly investigated the effects of the combination therapy on histological changes and fibrosis in a rat model of skeletal muscle fibrosis. Our results suggested that there was no inflammatory cell infiltration, and the shape of skeletal muscle cells was similar to the control group with the combination of massage and electroacupuncture therapy. Moreover, the combination of continuous massage and electroacupuncture therapy showed the least content of collagenous fiber compared with massage therapy or electroacupuncture therapy. The results suggested that the combination of continuous massage and electroacupuncture therapy manifested the optimal effect on histological changes and fibrosis. Next, we investigated the ultrastructure alteration of skeletal muscle in skeletal muscle fibrosis rats. Mitochondria in muscle physiology were regarded as the energy source by the generation of ATP [27]. Calcium ion uptake by mitochondria played a vital role in regulating calcium ion signal for the contraction-relaxation cycle in skeletal muscle [28]. The present study suggested that the combination of continuous massage and electroacupuncture therapy could restore the normal round or oval shape of mitochondria compared to the model group. Moreover, the combination of continuous massage and electroacupuncture therapy could also improve the arrangement of sarcomeres within one myofibril to be relative orderliness after blunt trauma to rat skeletal muscle.

Fibroblast and myofibroblast accumulation play an important role in the development of fibrosis, which can lead to produce the excessive extracellular matrix [29, 30]. The transdifferentiation of fibroblast to myofibroblast is regulated by the inflammatory factors [31]. Among the inflammatory factors, TGF-*β* is the primary factor that drives fibrosis in most, if not all [32]. TGF-*β* has three isoforms, including TGF-*β*1, TGF-*β*2, and TGF-*β*3. TGF-*β*1 was verified to induce myofibroblast differentiation and synthesis of extracellular matrix [33]. Our results suggested that either massage or electroacupuncture therapy could significantly downregulate the protein expression of TGF-*β*1 compared with the model group, with the strongest inhibition. Moreover, previous studies revealed that TGF-*β*1 could induce the expression of CTGF and *α*-SMA in fibroblasts [34]. CTGF acted as the important downstream factor of TGF-*β*1 and was an essential mediator for the composition of extracellular matrix, and *α*-SMA was a characteristic actin isoform expressed in myofibroblasts [35]. The combination of massage and electroacupuncture therapy could significantly downregulate the protein expression of CTGF compared with the model group. Furthermore, we found that the apoptosis of myofibroblast was induced in response to the combination of both massage and electroacupuncture. The increase of impaired matrix degradation in fibrosis can be reflected by decreased MMP-1 and increased TIMP-1, which is the inhibitor of MMP-1 [36]. The increased extracellular matrix leads to interstitial fibrosis by increasing the production of collagen type I and III. MMP-1 as the specific enzyme for collagen type I and III could help with the degradation of extracellular components. In contrast, TIMP-1, the inhibitor of MMP-1, inhibits collagen degradation. The dynamic change of MMP/TIMP is important for the degradation of extracellular matrix [37]. Our results suggested that the MMP-1 expression was downregulated, and the TIMP-1 expression was upregulated after blunt trauma to rat skeletal muscle, which resulted in downregulation of MMP-1/TIMP-1. This could destroy the balance of the synthesis and degradation for collagen type I and III and accelerate the fibrosis of injured skeletal muscle. The combination of massage and electroacupuncture therapy could reverse the unbalance of MMP-1/TIMP-1 induced by blunt trauma to rat skeletal muscle. These results suggested that the combination of massage and electroacupuncture therapy could reduce the inflammatory response and promote the MMP-1/TIMP-1 to degrade the excessive collagen type I and III and thus inhibit the fibrosis of injured skeletal muscle.

## 5. Conclusion

In the present study, the combination of massage and electroacupuncture therapy exhibited better effects than monotherapy on reducing skeletal muscle fibrosis in blunt trauma-induced rat skeletal muscle fibrosis. The combination of massage and electroacupuncture therapy could not only reduce the degree of fibrosis by downregulating the mRNA expressions and protein expressions of TGF-*β*1 and CTGF but also regulate MMP-1/TIMP-1 balance for extracellular matrix production. These results indicate that the combination of massage and electroacupuncture therapy has an additive effect on alleviating the fibrotic process.

## Data Availability

The datasets analyzed during the current study are available from the corresponding author on reasonable request.

## Supplementary Materials

Supplementary Materials Table S1: the real-time RT-PCR oligonucleotide primers.
