# Supplementary material for: The Combination of Electroacupuncture and Massage Therapy Alleviates Myofibroblast Transdifferentiation and Extracellular Matrix Production in Blunt Trauma-Induced Skeletal Muscle Fibrosis
Source: Evid Based Complement Alternat Med. 2021 Jul 7;2021:5543468. doi: 10.1155/2021/5543468 (PMC8282377; doi:10.1155/2021/5543468)
Supplement: Supplementary Materials — Table S1: the real-time RT-PCR oligonucleotide primers. [file 5543468.f1.docx]

Table S1. The real-time RT-PCR oligonucleotide primers.

| Gene | Primer | Sequence (5’-3’) | PCR product (bp) | |
| --- | --- | --- | --- | --- |
| GAPDH | Forward  Reverse | GTATGACTCTACCCACGGCAAGT  TTCCCGTTGATGACCAGCTT | 195 | |
| TGF-β1 | Forward  Reverse | TGGCTGAACCAAGGAGACGGAATA CACCTCGACGTTTGGGACTGATC | 118 | |
| CTGF | Forward  Reverse | GGGAAATGCTGTGAGGAGTGG  CATAGTTGGGTCAGGGCCA | 104 | |
| MMP-1 | Forward  Reverse | GACGTGGACCGACAACAGTGA  GGGGAACATTAGTGCTCCTACATC | 112 | |
| TIMP-1 | Forward  Reverse | GCAACTCGGACCTGGTTAT GTCGAATCCTTTGAGCATCTT | 113 | |
|  | | | |  |
